# Pharmacokinetics of Mycophenolate Mofetil and Development of Limited Sampling Strategy in Early Kidney Transplant Recipients

**DOI:** 10.3389/fphar.2018.00908

**Published:** 2018-08-13

**Authors:** Jun Zhang, Zhi Sun, Zhenfeng Zhu, Jing Yang, Jian Kang, Guiwen Feng, Lin Zhou, Lihua Zuo, Yonggang Luo, Xiaojian Zhang

**Affiliations:** ^1^Department of Pharmacy, The First Affiliated Hospital of Zhengzhou University, Zhengzhou, China; ^2^Department of Renal Transplantation, The First Affiliated Hospital of Zhengzhou University, Zhengzhou, China; ^3^Department of Integrated Intensive Care Unit, The First Affiliated Hospital of Zhengzhou University, Zhengzhou, China

**Keywords:** mycophenolate mofetil, mycophenolic acid, pharmacokinetic, limited sampling strategy, renal transplantation

## Abstract

The mycophenolate mofetil (MMF) dose management for optimization of post-transplant treatment especially the early postoperative phase has been well recognized. MMF is a pro-drug of mycophenolic acid (MPA) and is widely used in Chinese renal transplant patients. Until now, the pharmacokinetic (PK) characteristics and model for the area under the concentration–time curve for the 12-h (h) of exposure (AUC_0-12__h_) of MPA (MPA-AUC_0-12__h_) estimation were lacking for the new formulation of MMF dispersible tablet in renal transplant patients. The aims of the study were to investigate the PK characteristics of MMF dispersible tablet by detecting the active metabolite of MPA and to establish an accuracy and precision equation for calculating MPA-AUC_0-12__h_ by limited sampling strategy (LSS) in Chinese kidney transplant patients. A total of 60 postoperative kidney transplant recipients were given a multiple-dose of MMF dispersible tablet twice daily combination with tacrolimus (Tac) and steroids. On the 5th day post-transplantation, blood specimens were collected before drug administration and up to 12 h after MMF dispersible tablet administration. Non-compartmental PK analysis was used to determine the data obtained from individual patients. Multivariate stepwise regression analysis was used to develop models for predicting MPA-AUC_0-12__h_. The 3- and 4-point sampling models using 2 h, 4 h, 8 h and 1 h, 2 h, 4 h and 8 h, respectively, allowed accurate estimation of MPA-AUC_0-12__h_. PK parameters of MMF dispersible tablet were obtained and the 4-point LSS is the best model for accurate and precise estimation of MPA-AUC_0-12__h_.

## Introduction

**FIGURE 1:**
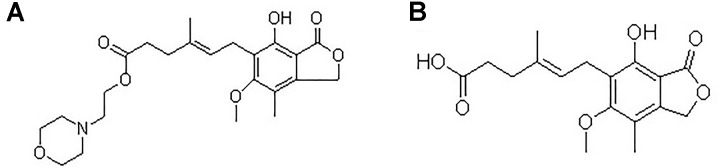
Chemical structures of MMF **(A)** and MPA **(B)**.

Mycophenolate mofetil (MMF), an ester prodrug of mycophenolic acid (MPA), is the first-line immunosuppressant drug used with other drugs in prevention and treatment of graft rejection in solid organ transplantation. Chemical structures of MMF and MPA are shown in **Figure 1**. Dispersible tablet a new formulation of MMF is widely used in renal transplant patients in China. As MPA has a narrow therapeutic window and large inter-individual variability (Staatz and Tett, 2007; Kuypers et al., 2010; Mathew et al., 2010; Saint-Marcoux et al., 2011), therapeutic drug monitoring (TDM) of the area under the concentration–time curve for the 12-h (h) of exposure (AUC_0-12__h_) of MPA (MPA-AUC_0-12__h_) is essential to improve clinical outcomes (van Gelder and Shaw, 2005; de Winter et al., 2007; West-Thielke and Kaplan, 2007). Adequate MPA exposure is especially important to prevent acute rejection in the early renal transplant phase, especially the 1st week after transplantation (Tett et al., 2011; Barraclough et al., 2012b). The MPA-AUC_0-12__h_ has been recommended as the best marker for the MMF dose adjustment to achieve optimal efficacy and keep the toxicity to minimum in clinical application. However, increasing studies reported that the trough level (*C*_0_) of MPA showed a poor correlation with the MPA-AUC_0-12__h_ (Mathew et al., 2010; Barraclough et al., 2012a; Chaabane et al., 2013; Cai et al., 2015). Owing to the requirement for frequent blood sampling, full MPA-AUC_0-12__h_ monitoring is laborious. The limited sampling strategy (LSS), using a limited number of blood samples to estimate the MPA-AUC_0-12__h_ (Baraldo et al., 2009; Barraclough et al., 2012a), solves this problem. The two formulations of MMF (dispersible tablet and capsule) were bioequivalent in healthy Chinese volunteers (Zhang et al., 2010). Due to the differences in the formulations of capsule and dispersible tablet, the PK of MPA may be different. The dynamic profile of the new MMF dispersible tablet in Chinese kidney transplant recipients is unclear. With the increasing use of MMF in renal transplant patients in China, the need for concentration-controlled dosing, based on MPA PK characteristics of MMF dispersible tablet, is becoming important in order to promote optimum safety and efficacy. It is of clinical importance to obtain a better understanding of the dynamic characters of MMF dispersible tablet. Until now, PK data of MPA, based on the new formulation of MMF was lacking in Chinese renal transplant patients. Meanwhile, there was no LSS of MMF dispersible tablet has been suggested for Chinese kidney transplant recipients. So, the accurate and convenient LSS of MMF dispersible tablet for predicting MPA-AUC_0-12__h_ in renal transplant recipients is needed to be developed based on the dynamic characters of the new formulation.

The aims of the study were to investigate the PK characteristics of MPA in Chinese kidney transplant patients taking MMF dispersible tablet in association with steroids and tacrolimus (Tac) and to develop an accuracy and precision LSS for MPA-AUC_0-12__h_ calculation in large number of samples in such patients.

## Materials and Methods

### Patients Selection

Consecutive, prospective 60 patients were included in the study. All the patients were over 18 years and underwent the first kidney transplant in this single-center study. Patients were recruited after passing a physical examination and laboratory tests, which included blood biochemistry, hematology, and urine analysis. We excluded patients with cancer, patients with hematologic abnormality, hepatic abnormality, gastrointestinal disorder, pregnancy, systemic infection, or any acute disease, and patients who allergy to MMF dispersible tablet, MPA, steroids or Tac. All patients underwent the same therapeutic scheme of MMF dispersible tablet with Tac and steroids as concomitant immunosuppressive therapy. This research was conducted according to the principles of the Declaration of Helsinki (1964) and its later amendments. The clinical protocol and the informed-consent form were approved by the Ethics Committees of The First Affiliated Hospital of Zhengzhou University. All participants were informed of the details and procedures of the research before they signed a written informed consent.

### Study Design

The study was carried out in two steps. We enrolled 40 patients in the first step as the model-building group. The model-building group was used to establish optimal LSS for predicting AUC of MPA.The 20 patients who were enrolled later, were used as the validation group. The validation group was used to assess the accurate and practical applicability of the optimal LSS. The study was carried out in the Clinical Nephrotransplantation Center of the First Affiliated Hospital of Zhengzhou University.

The renal transplant recipients received 500 or 750 mg of MMF dispersible tablet (250 mg/Tab) twice daily with water. The starting Tac dose was 0.1–0.15 mg/kg/day. Targeted Tac whole-blood trough concentration was 8–15 ng/mL and the Tac concentration was assayed by enzyme-multiplied immunotechnique. All patients received two intraoperative corticosteroid of 500 mg of methylprednisolone. Maintenance methylprednisolone dose was tapered to 500 mg on day 2, followed by a stepwise reduction to 375 mg on day 3, 250 mg on day 4, and 120 mg on day 5. Then methylprednisolone (16 mg/d) was administered. Patients fasted overnight before dosing and given standardized meals until 1.5 h after MMF dispersible tablets administration, serial blood samples (2 mL each) were drawn in vials containing EDTA at 0, 0.5, 1, 1.5, 2, 3, 4, 6, 8, 10, and 12 h (*C*_0_, *C*_0.5_, *C*_1_, *C*_1.5_, *C*_2_, *C*_3_, *C*_4_, *C*_6_, *C*_8_, *C*_10_, and *C*_12_) after the oral morning dose of MMF dispersible tablet on the 5th day after transplantation. The samples were sent to lab on ice. The blood samples were centrifuged at 3000 r/min and plasma samples were separated. MPA concentrations were analyzed using the validated ultra-performance liquid chromatography ultraviolet (UPLC-UV) method (Zhang et al., 2017).

### Non-compartmental PK Parameters Analysis

The PK analysis was performed using WinNonlin version 7.0 (Pharsight Corporation, Mountain View, CA, United States). Non-compartmental PK analysis was used to determine the data obtained from individual patients. The area under the concentration-time curve 0 to time (AUC_0-t_) calculated by linear trapezoid method. Predose concentration (*C*_0_), post-dose minimum and peak plasma concentration (*C*_min_ and *C*_max_), time to reach *C*_max_ (*T*_max_) were obtained directly from the concentration vs. time profiles. Total body clearance (CL) was calculated by dose/AUC_0-τ_ (τ was administration interval). Given the difference in doses, we normalized the AUC_0-12__h_ of 500–750 mg. Dose-normalized estimate of AUC_0-12__h,norm_ was derived by the corresponding value dividing the result that 750 mg divided 500 mg. Inter-individual variability was assessed using coefficient of variation (CV).

### Development of LSSs

The study had sufficient sampling points to capture the full characteristics of MPA’s PK. Since, to date, model for estimation of MPA-AUC_0-12__h_ was lacking for MMF dispersible tablet in Chinese renal transplant patients. The time control plasma concentrations of MPA would be the most sensitive to the differences in the drug absorption rate, distribution volume and elimination rate. So, the LSS was performed to estimate MPA-AUC_0-12__h_. The data were divided into a model-building group (*n* = 40) and a model validation group (*n* = 20). All results were presented as mean ± SD. The data were performed using SPSS version 18.0 (SPSS Inc., Chicago, IL, United States). Step 1: the fAUC_0-12__h_ was calculated by linear trapezoid method. Prediction equation models were derived by multiple regression analysis, using MPA concentrations at different time points. The fAUC_0-12__h_ as a dependent variable and the concentration of MPA at each time point were used as independent variable. The correlation coefficient (*R*^2^) was used to evaluate the regression level of the equation. The fAUC_0-12__h_ was compared with eAUC_0-12__h_ calculated using the selected equation of Step 1 in the validation group. The prediction bias were quantified as the percentage of mean prediction error (MPE%) and mean absolute prediction error (MAPE%). The two error parameters were calculated by the following equations:

(1) $$MPE\%=\frac{1}{n}\sum_{i=1}^{n}\{({\text{eAUC}}_{0-12\;\text{h}}-{\text{fAUC}}_{0-12\;\text{h}})/{\text{fAUC}}_{0-12\;\text{h}}\}\times 100$$

(2) $$MAPE\%=\frac{1}{n}\sum_{i=1}^{n}\{|({\text{eAUC}}_{0-12\;\text{h}}-{\text{fAUC}}_{0-12\;\text{h}})/{\text{fAUC}}_{0-12\;\text{h}}|\}\times 100$$

The Bland–Altman test (Bland and Altman, 1986) was performed to assess the consistency (bias and precision) between the fAUC_0-12__h_ and eAUC_0-12__h_ in the validation group. Bias and precision were visualized by plotting the average AUC_0-12__h_ that results from the estimated and full profile. The eAUC_0-12__h_ was calculated using the selected equation. The correlation between fAUC_0-12__h_ and eAUC_0-12__h_ was expressed by scatter diagram. The best model was selected based on account the values of *r*^2^, predictive bias, and the Bland–Altman analysis.

### Tolerability Evaluation

The investigators including clinicians and clinical pharmacists who monitored the patients for adverse events (AEs). The primary outcomes were 1-week incidence of acute rejection, graft function and other AEs during hospitalization. Delayed graft function was defined as dialysis requirement in the 1st week post-transplantation. Secondary outcomes included 6-month incidence of acute rejection, graft survival and other related AEs. Those events were also determined using patient interview (subjective complaints by patients, objective observation by clinicians) regarding the potential presence of AEs, such as diarrhea, nausea and so on, at any time during hospitalization and by comparing the results of laboratory tests (hematology, blood biochemistry, hepatic function and urinalysis), during the study and 6 months after completion of the study (follow-up visit).

### Statistical Analyses

Differences in efficacy and safety end points were evaluated by the Chi-square or Fisher’s exact test. The correlations between AEs and covariates (age, body weight, MPA-AUC_0-12__h_) were evaluated by Spearman test. All statistical analyses were performed with SPSS version 18.0. *P* < 0.05 was considered statistically significant.

## Results

### Patients

A total of 60 patients were enrolled in the study. **Table 1** summarizes the baseline characteristics of the patients.

**Table 1:** The demographic data and clinical parameters.

Parameter	Study group	Validation group
Gender (male/female)	40 (32/8)	20 (15/5)
Age (years)	34.10 ± 9.13	32.95 ± 11.12
Race (Han/other)	40 (40/0)	20 (20/0)
Body weight (kg)	64.11 ± 9.40	60.78 ± 8.02
Serum creatinine (μmol/L)	139.5 ± 93.53	110.25 ± 32.89
Alanine transferase (U/L)	15.75 ± 9.96	12.75 ± 7.45
Aspartate transferase (U/L)	15.08 ± 8.08	11.95 ± 4.05
Albumin (g/L)	40.9 ± 5.47	39.64 ± 4.32
Total bilirubin (μmol/L)	9.6 ± 6.76	13.05 ± 5.67
Tac C_0_ (ng/mL)	11.85 ± 2.40	11.71 ± 3.17

### Non-compartmental Analysis

**FIGURE 2:**
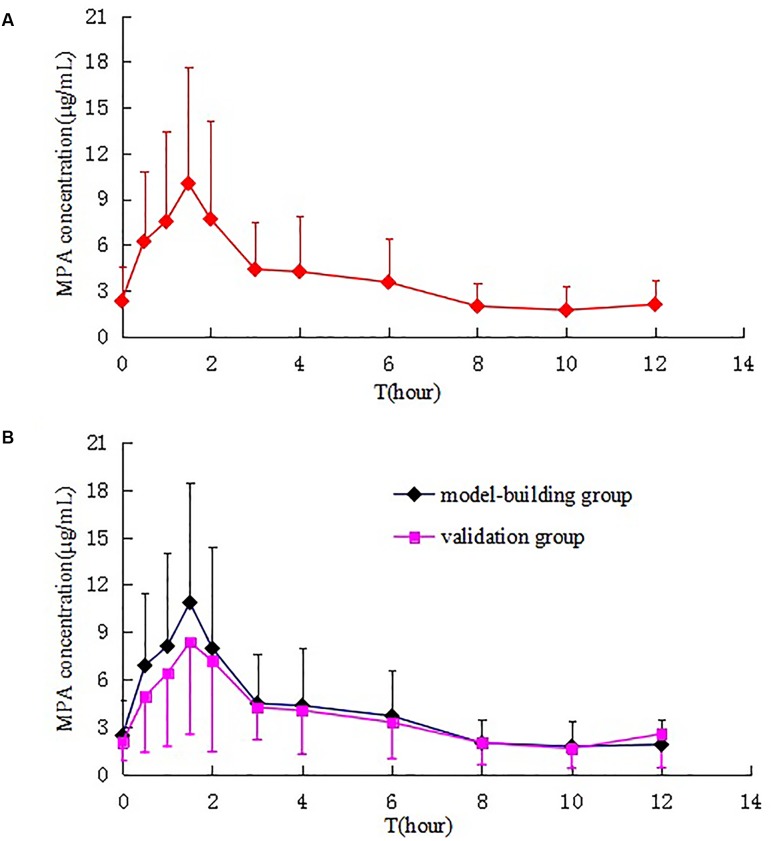
Mean plasma concentration–time curves of MPA after administration of MMF dispersible tablets in all patients **(A)**, model-building group and validation group **(B)**.

A total of 60 patients participated in the PK study. The mean plasma concentration–time curves of MPA after administration of MMF dispersible tablets in all patients, model-building group and validation group are shown in **Figure 2**. The MPA plasma PK parameters are summarized in **Table 2**. Inter-individual variability (CV) in AUC_0-12__h_, *C*_max_, *C*_min_, *C*_0_, CL, and *T*_max_, respectively, were 15.19, 27.97, 89.72, 66.53, 23.56, and 80.78%. Due to the enterohepatic recirculation (EHR) most of the patients revealed a second small peak in the 4–12 h after taking MMF dispersible tablets.

**Table 2:** Pharmacokinetic (PK) parameters of MPA after multiple dose of MMF dispersible tablet in 60 kidney transplant recipients.

Parameters	Model-building group	Validation group	All patients
AUC_0-12__h_ (μg⋅h/mL)	47.08 ± 22.26	42.13 ± 14.99	45.43 ± 20.14
AUC_0-12__h,norm_ (μg⋅h/mL)	49.14 ± 21.78	42.13 ± 14.99	46.81 ± 19.93
*C*_min_ (μg/mL)	1.31 ± 1.09	1.16 ± 1.01	1.26 ± 1.06
*C*_max_ (μg/mL)	13.81 ± 7.94	11.36 ± 5.92	13.00 ± 7.37
*C*_0_ (μg/mL)	2.44 ± 2.21	2.02 ± 1.17	2.30 ± 1.93
*T*_max_ (h)	1.46 ± 0.76	1.75 ± 1.15	1.56 ± 0.91
CL (L/h)	17.92 ± 8.01	19.12 ± 6.40	18.32 ± 7.48

### Results of LSSs

The results of regression equations obtained in the study group between each sampling time point and the fAUC_0-12__h_ are shown in **Table 3**. The equation containing only the trough level showed a weak correlation (*R*^2^ = 0.501) with fAUC_0-12__h_.

**Table 3:** Univariate correlation between the MPA plasma concentration at each time point and the AUC_0-12__h_.

Time	Equation	*R*^2^	SE of estimation
*C*_0_	*y* = 29.71 + *C*_0_ × 7.126	0.501	15.93
*C*_0.5_	*y* = 36.47 + *C*_0.5_ × 1.546	0.103	21.36
*C*_1_	*y* = 31.46 + *C*_1_ × 1.93	0.267	19.31
*C*_1.5_	*y* = 31.99 + *C*_1.5_ × 1.39	0.225	19.86
*C*_2_	*y* = 28.85 + *C*_2_ × 2.29	0.436	16.94
*C*_3_	*y* = 23.54 + *C*_3_ × 5.27	0.538	15.33
*C*_4_	*y* = 29.86 + *C*_4_ × 3.97	0.427	17.07
*C*_6_	*y* = 33.01 + *C*_6_ × 3.83	0.242	19.63
*C*_8_	*y* = 22.61 + *C*_8_ × 12.47	0.682	12.71
*C*_10_	*y* = 29.32 + *C*_10_ × 10.15	0.548	15.16
*C*_12_	*y* = 30.01 + *C*_12_ × 9.19	0.453	16.68

**FIGURE 3:**
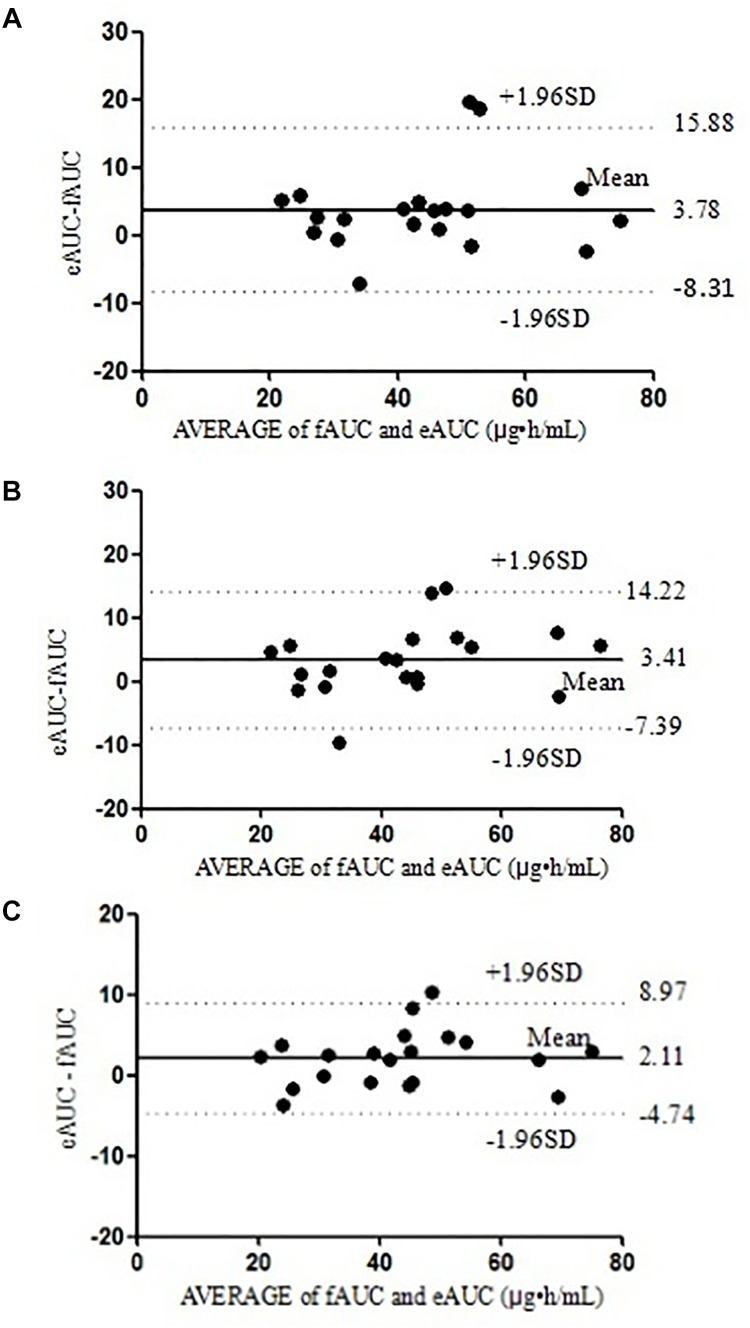
The Bland–Altman analysis agreement between the fAUC and eAUC using model 2 **(A)**, model 3 **(B)**, and model 4 **(C)**.

**FIGURE 4:**
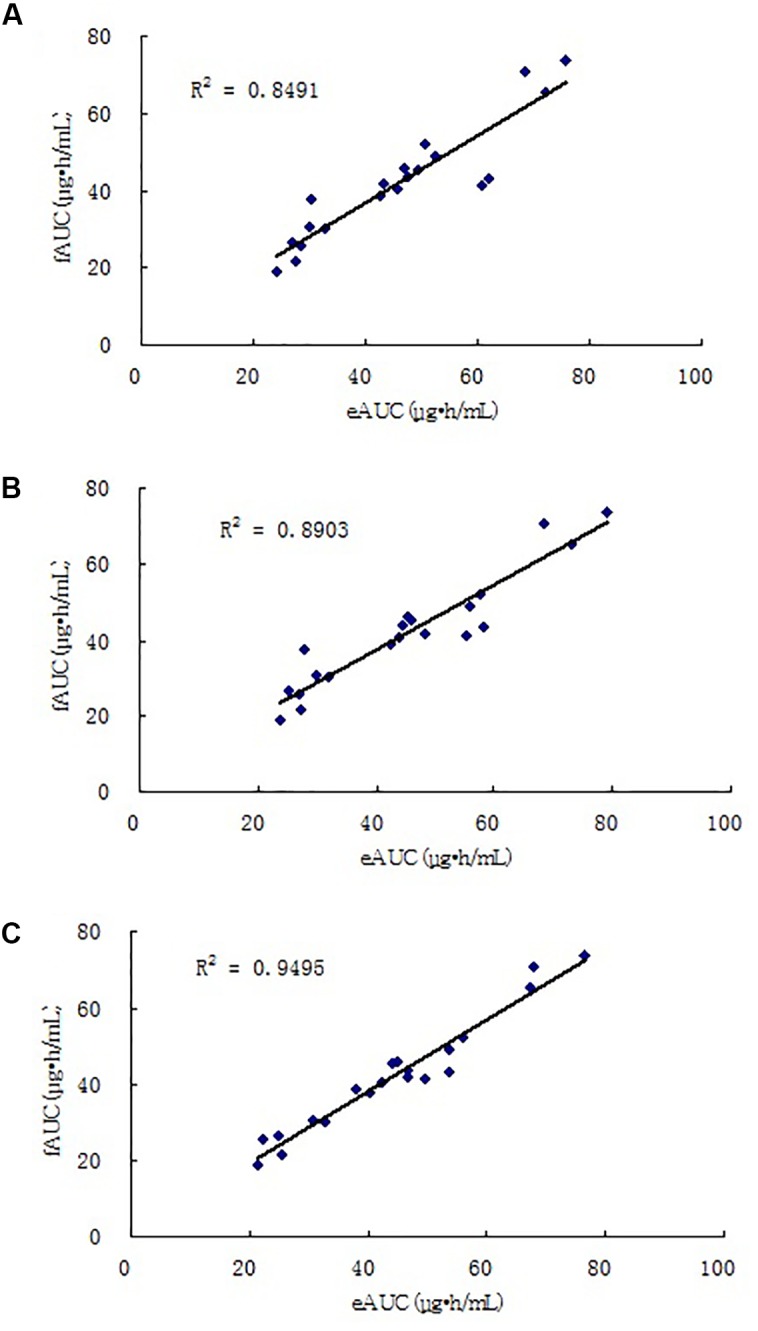
Correlations between eAUC and fAUC calculated using model 2 **(A)**, model 3 **(B)**, and model 4 **(C)**.

Equations for two-, three-, and four-point estimations of the MPA-AUC_0-12__h_ were obtained by multiple stepwise regression analysis. As seen in **Table 4**, with the number of sampling time points increasing, the linear correlations between the eAUC_0-12__h_ and fAUC_0-12__h_ also improved. Using the three selected equations obtained in the model building group, we compared the differences between eAUCs and fAUCs in the model validation group. The predictive results are shown in **Figures 3**, **4**. All the three models were found to have good mean residuals and symmetries.

**Table 4:** Multiple linear regression analysis of the AUC_0-12__h_.

Model	Time	Equation	*R*^2^	SE of estimation
1	*C*_8_	*y* = 22.612 + *C*_8_ × 12.467	0.682	12.71
2	*C*_8_, *C*_2_	*y* = 14.04 + 10.43 ×*C*_8_ + 1.58 ×*C*_2_	0.870	8.20
3	*C*_8_, *C*_2_, *C*_4_	*y* = 11.95 + 8.9 ×*C*_8_ + 1.41 ×*C*_2_ + 1.48 ×*C*_4_	0.910	6.76
4	*C*_8_, *C*_2_, *C*_4_, *C*_1_	*y* = 8.36 + 7.49 ×*C*_8_ + 1.34 ×*C*_2_ + × 1.66 ×*C*_4_ + 0.76 ×*C*_1_	0.948	5.34

The differences between the eAUCs obtained with 2, 3, or 4 points and the fAUCs in the 20 validation patients were 3.78 ± 6.17, 3.41 ± 5.51, and 2.11 ± 3.50 μ⋅h/mL, respectively; 95% confidence intervals were (-8.31 to 15.88), (-7.39 to 14.22), and (-4.74 to 8.97), respectively.

For the two-point equation, the MPE% and MAPE% values were 10.28 and 12.99%, respectively. The mean values of fAUC and eAUC were not significantly different (42.13 ± 14.99 μg⋅h/mL and 45.92 ± 15.86 μg⋅h/mL, respectively). The agreements between fAUCs and eAUCs were tested by Bland–Altman Analysis, 10% (2/20) of patients were outside the 95% confidence interval (**Figure 3A**).

For the three-point model, the MPE% and MAPE% values were 8.64 and 12.39%, respectively. The mean values of fAUC and eAUC were not significantly different (42.13 ± 14.99 μg⋅h/mL and 45.55 ± 16.54 μg⋅h/mL, respectively). The Bland–Altman Analysis confirmed the agreement between eAUCs and fAUCs, and 5% (1/20) of patients were outside the 95% confidence interval (**Figure 3B**).

The four-point model, the MPE% and MAPE% values were 5.26 and 8.35%, respectively. The mean values of fAUC and eAUC were not significantly different (42.13 ± 14.99 μg⋅h/mL and 44.24 ± 15.54 μg⋅h/mL, respectively). The Bland–Altman Analysis showed the best agreements between the eAUCs and fAUCs, and 5% (1/20) of patients were outside the 95% confidence interval (**Figure 3C**). The abilities of the three models to predict the MPA–AUC_0-12__h_ are depicted in **Figure 4**. Based on the above analysis, the 4-point method (*C*_8_, *C*_2_, *C*_4,_ and *C*_1_) was the best LSS.

### Tolerability

No serious AEs occurred in the study. There were no patients who suffered from acute rejection, suspected acute rejection, delayed graft function or systemic infection during hospitalization and 6 months post-transplantation. Results of vital signs, and physical examinations were within normal limits for all participants during hospitalization. The Spearman correlations between AEs and age, body weight or MPA-AUC_0-12__h_ were 0.29, 0.24, and 0.12, respectively. Factor that significantly influenced the incidence of AEs in the Spearman test was MPA-AUC at day 5 (*P* = 0.023). The incidences of AEs in different MPA-AUC_0-12__h_ ranges were listed in **Table 5**. There were no significant differences in the incidences of AEs between the patients with MPA-AUC_0-12__h_ levels no more than 60 μg⋅h/mL and more than 60 μg⋅h/mL at the 5 days after transplantation. This may related to the small sample size. Though 60 μg⋅h/mL was not a useful threshold, a relationship was still seen between AUC and adverse effects. Mean MMF daily doses at discharge were similar with hospitalization. Two patients suffered constipation at 2 months post-transplantation. All these AEs were mild. The formulation was well tolerated in transplant patients in the study.

**Table 5:** Comparison of incidences of AEs in different MPA-AUC_0-12__h_ ranges.

	MPA-AUC_0-12__h_ ≤ 60 μg⋅h/mL at Day 5 (*n* = 49)	MPA-AUC_0-12h_ > 60 μg⋅h/mL at Day 5 (*n* = 11)	*P*
Diarrhea	17 (34.7)	3 (27.3)	0.74
Vomiting	8 (16.3)	4 (36.4)	0.21
Eructation	12 (24.5)	4 (36.4)	0.46
Nausea	9 (18.4)	3 (27.3)	0.68
Anemia	10 (20.4)	5 (45.5)	0.12
Tremor	1 (2.0)	0 (0.0)	0.10

## Discussion

The MMF dose management for optimization of post-transplant treatment especially the early postoperative phase has been well recognized. PK studies in the renal transplant population are essential to determine the correct dosage strategies for optimal efficacy. There is an increasing interest in use of MMF dispersible tablet in Chinese renal transplant patients. To date, most studies investigating the PK of MPA in this population applied a sparse sampling protocol, which may have limited the overall precision of the final model. In the present study, the full PK character was available for all the patients and can be used to provide limited sampling strategies information for the early transplant phase patients. The PK characteristics of MMF and equation for estimation of MPA-AUC_0-12__h_ were lacking for the new formulation of MMF in Chinese renal transplant patients. The present study developed LSSs of MMF dispersible tablet in renal transplant patients for the first time.

As previous studies reported, the PK of MPA showed a large interpatient variability (van Hest et al., 2005; Staatz and Tett, 2007; Kiang and Ensom, 2017; Yu et al., 2017). In our study after oral MMF dispersible tablet, the *t*_max_ occurred at 0.5 to 6 h after MMF dispersible tablet dosing (mean peak time point: 1.5 h post-dose) and *C*_max_ ranged from 4.26 to 38.4 μg/mL. The interindividual variability in AUC_0-12__h_, *C*_max_, *C*_min_, *C*_0_, CL, and *T*_max_ were large. Previous studies (Le Guellec et al., 2004; Yu et al., 2017) found that BW was positively correlated with CL of MPA in adult transplant patients. Kagaya et al. (2017) reported similar results that a lower BW was independently predictive for a higher MPA-AUC_0-12__h_. CL of MPA was found significant time-dependency in previous study (van Hest et al., 2007). The CL of this study was 18.32 L/h, which was similar to those previously reported in adult kidney transplant recipients (de Winter et al., 2011; Yu et al., 2017). When given 750 mg twice daily, most patients can reach the target exposure in 1st week after renal transplantation.

Mycophenolic acid is primarily metabolized to the inactive metabolite 7-*O*-MPA glucuronide, which undergoes EHR (Armstrong et al., 2005). Due to EHR a second peak was identified in 6–12 h following MMF oral administration (Bullingham et al., 1998). However, the secondary concentration peaks were relatively small in most of our patients. The reason for little EHR is likely due to the weak continuous secretion of bile into the small intestine. Less 7-O-MPA glucuronide was deglucuronidated in the gut and re-entered the circulation as MPA. The enzyme-multiplied immunoassay technique (EMIT) and UPLC are two principal methods used to monitor MPA concentration. The UPLC-UV method is highly specific for determination of MPA and has low imprecision across the analytical range. In comparison with UPLC, EMIT contributed to a larger measurement errors due to its cross-reaction with acyl-MPA glucuronide (Yu et al., 2017). UPLC method used in this study is an ideal method for rapid detection with high accuracy, and good reproducibility.

The present study shows that the interindividual variability is large, which highlight the TDM of MPA in renal transplant patients. An appropriate AUC_0-12__h_ after drug administration, between 30–60 μg⋅h/mL, was associated with significant decrease in acute graft rejection in kidney transplant patients (Staatz and Tett, 2007). TDM of MPA plays an important role in performing personalized prescription for the prognosis of recipients. The LSS technique has been applied in estimating the MPA exposure of MMF capsule in the past decades. For MMF capsule, substantial research has shown that an LSS can predict MPA-AUC_0-12__h_ in different populations after transplantation (Kuypers et al., 2010; Mathew et al., 2010; Saint-Marcoux et al., 2011; Chaabane et al., 2013). However, those models cannot accurately estimate the AUC values of MMF dispersible tablet in most of our patients. This result may relate to the different PK profiles of MMF dispersible tablet with MMF capsule in Chinese population. MMF dispersible tablet lead a new PK profile which makes it different with MMF capsule in selecting the optimal model for TDM. So, special LSS models are needed for the new formulation of MMF to guide the clinical pharmacists and clinicians to scheme medication regimens for patients. Considering the huge PK variability of MPA, we enrolled 40 patients in the model-building group to establish more practical and precision models. Then we performed external validation with another 20 patients to verify the practicability of the model.

The MPA PK profile is affected by the associated calcineurin inhibitors (Atcheson et al., 2005; Barraclough et al., 2010). The cyclosporine-based studies have been reported to result lower total MPA exposure compared with the Tac-based regimen (Filler et al., 2000). So, in this study, we enrolled the Tac-based regimen patients. The association between the trough level of MPA and clinical outcome such as graft rejection is still controversial. Some researches have shown that the trough level of MPA may be correlated to the patient’s outcomes in kidney transplant recipients (Borrows et al., 2006; Miura et al., 2011). However, many studies have indicated a poor correlation between single-point concentrations and MPA-AUC_0-12__h_ (Barraclough et al., 2012a; Chaabane et al., 2013; Yamaguchi et al., 2013; Cai et al., 2015; Jia et al., 2017). The present study also revealed the same result. The *C*_0_ level was not a reliable parameter to assess the MPA exposure (*R*^2^ = 0.501). The best correlations between the single-point concentration and fAUC occurred at 3 h (*R*^2^ = 0.538), 8 h (*R*^2^ = 0.682), and 10 h (*R*^2^ = 0.548) treatment with MMF dispersible tablet during the 1st week after kidney transplantation. These results are different with that found by the previous study (*R*^2^ < 0.4 at any single-point concentration) in Chinese patients treated with MMF capsule in combination with Tac and steroids (Cai et al., 2015). Then, we used the stepwise analysis obtained the equations were listed in **Table 4**. In the past reports, there were also no models which include single sample concentration could accuracy predict the fAUC of MPA in renal transplant recipients (Pawinski et al., 2002; Baraldo et al., 2009; Miura et al., 2009; Yamaguchi et al., 2013; Cai et al., 2015). As a result, two time points (*C*_2_ and *C*_8_), three time points (*C*_2_, *C*_4_, and *C*_8_) and four time points (*C*_1_, *C*_2,_ *C*_4_, and *C*_8_) models were developed in this study. The frequency of sampling times was acceptable in all models.

In this study, the two-sample equation displayed a good agreement with the fAUC. The significant correlation for predicting MPA-AUC_0-12__h_ was *C*_2_ and *C*_8_ with acceptable MPE% and MAPE%. This is the best practicability model with the least blood samples based on the dynamic characters of MMF dispersible tablet in this study. However, in the literature, there were no two-sample models to predict the MPA-AUC_0-12__h_ in early kidney transplant recipients were recommended for clinical practice.

Compared with the two-sample model the 3-time point equation was better correlate with the fAUC. Our equation was different with the published three-point combinations (*C*_0_/*C*_1_/*C*_4_, *C*_2_/*C*_6_/*C*_10_, *C*_2_/*C*_4_/*C*_9_, *C*_0_/*C*_1_/*C*_2_, and *C*_0_/*C*_0.5_/*C*_2_) (Pawinski et al., 2002; Muller et al., 2007; Miura et al., 2008; Chaabane et al., 2013; Cai et al., 2015). In the previous published three-point models (*C*_2_/*C*_6_/*C*_10_) (Chaabane et al., 2013) the authors choose the patients on 7 days after transplantation. The other models (*C*_0_/*C*_1_/*C*_4_, *C*_2_/*C*_4_/*C*_9_, *C*_0_/*C*_1_/*C*_2_, and *C*_0_/*C*_0.5_/*C*_2_) (Pawinski et al., 2002; Muller et al., 2007; Miura et al., 2008; Chaabane et al., 2013) the patients were on the steady state period. The main reasons that lead to the differences between the published LSSs with our model (*C*_2_/*C*_4_/*C*_8_) possibly were the patient ethnicity, number of patients, pathological conditions and formulation of MMF.

In this study, the best model to assess the fAUC as tested by Bland–Altman analysis was 8.36 + 7.49 ×*C*_8_ + 1.34 ×*C*_2_ + × 1.66 ×*C*_4_ + 0.76 ×*C*_1_ (*R*^2^ = 0.948). This equation was based on four time points provided the most reliable estimation of the MPA-AUC_0-12__h_. The model included absorption phase, distribution phase and elimination phase after the MMF dispersible tablet administration.

Considering the MPA kinetic characters that investigated in the present research, three model equations were suggested with strong correlations between the fAUCs and eAUCs. The long time span may restrict the models application for the outpatients, but it was a useful tool to guide the individualization prescription of MMF dispersible tablet dose for the inpatients, especially for early post-transplant phase. The best model was selected by taking into account the values of *r*^2^, predictive bias, and the Bland–Altman analysis. The 4-point method (*C*_8_, *C*_2_, *C*_4_, and *C*_1_) was the best LSS. This model can be recommended for predicting MPA-AUC_0-12__h_ in early renal transplant recipients in clinical practice.

## Conclusion

In this study, the PK characteristics of MMF dispersible tablet was studied in the early renal transplant patients. The result revealed new insights into the PK characters of MPA after multiple dose of MMF dispersible tablet. Large interindividual variability in MPA PK was demonstrated. The accurate and clinically feasible models for evaluating MPA exposure level in renal recipients after administration of MMF dispersible tablet, Tac and steroids were developed. This information can help to apply TDM of MPA more efficient. The 4-time point model was the best LSS. The applicability of this model in predicting MPA-AUC_0-12__h_ had been validated in early Chinese kidney transplant populations. The finding can be recommended in early Chinese adult renal transplant recipients to individualize the dosage regimen in clinical application.

## Author Contributions

JZ, ZS, YL, and XZ designed the research. ZZ, JK, and GF performed the experiments. JY, JZ, LZ, and LhZ analyzed the results. All authors approved the final manuscript.

## Conflict of Interest Statement

The authors declare that the research was conducted in the absence of any commercial or financial relationships that could be construed as a potential conflict of interest. The reviewer DM and handling Editor declared their shared affiliation.
